# Work, boredom and rhythm in the time of COVID-19

**DOI:** 10.1177/00380261221147749

**Published:** 2023-01-19

**Authors:** Louise Nash, Dawn Lyon

**Affiliations:** University of Essex, UK; University of Kent, UK

**Keywords:** acedia, boredom, homeworking, pandemic, rhythmanalysis

## Abstract

This article uses Henri Lefebvre’s *Rhythmanalysis* as a foundational text for researching boredom, and offers a critical analysis of UK-based media commentaries about boredom and homeworking written during 2020 and 2021. We situate the discussion within the rhythmic rupture caused by the COVID-19 pandemic and foreground rhythm as a lens for understanding reported experiences and reflections on boredom and work. For non-essential workers, lockdown offered an opportunity to reconfigure working lives away from the constraints of commutes and everyday work settings, yet our findings highlight the narrative representation and experience of a particular type of boredom and inertia known as acedia. The analysis discusses the presence of acedia and absence of rhythm across three themes: acedia and being stuck in time and space; embodiment, movement and rhythm; and the relationship between the present and the future. We conclude by considering what the experience of boredom might mean for how we reconceptualise our post-pandemic working lives.

## Introduction

Whilst boredom is taken for granted as a common experience of modern life (Goodstein, 2005; Healy, 1984), it has received limited sociological attention. In recent debates, it has been recognised as socially constructed and context specific rather than a universal feature of human existence, and appreciated as a phenomenon associated with modernity (Carroll et al., 2010; Costas & Kärreman, 2016; Johnsen, 2016). In his *Introduction to Modernity* (1995), sociologist, philosopher and literary and urban scholar Henri Lefebvre views the phenomenological lived experience of boredom as both a reaction to and a consequence of modernity, symptomatic of deeper social currents (Gardiner, 2012). The artificial regulation of time in modernity and associated bureaucracy are connected to the rise of boredom (Anderson, 2004; Johnsen, 2016). In existing literature, boredom is variously understood as arising from the person (ongoing work in psychology continues to discuss ‘boredom-proneness’, e.g. Boylan et al., 2021) or the situation (especially work, e.g. Roy, 1959). It is widely conceived as an ‘absence’ in opposition to the ‘interesting’ (see Anderson, 2004), as overload or lack of stimulation, and explored for its potential both as disenchantment and the possibility of it leading to re-enchantment with the world (Mosruinjohn & Matorina, 2019–2020). Boredom has been considered by philosophers and artists as part of a cultural mood (Dag Holmboe & Morris, 2021; Highmore, 2010; Mosruinjohn & Matorina, 2019–2020), yet in the social sciences, ‘boredom studies’ have been dominated by quantitative questionnaire-based psychological research which does not capture its dynamic and symbolic qualities (Gardiner & Haladyn, 2016).

In this article, we contend that boredom – and especially boredom at/in work – can be fruitfully explored by bringing it into dialogue with the concept of rhythm. Boredom is associated with time in terms of quantity – too little, too much – and quality – too busy, too empty – resulting in an endless present (Loukidou et al., 2009) as well as space (Gamsby, 2018). Rhythm also attends to the interplay of time and space in everyday life across embodied, affective and cognitive dimensions. Pointing to the relevance of rhythm in understanding boredom, Paasonen argues for the recognition of ‘shifting intensities and qualities of experience – as in the cycles of “bored, not bored, bored, not bored, bored”’ (2022, p. 151). Rhythm, we contend, offers a tool for disentangling different dimensions or qualities of boredom. Yet the relationship between boredom and rhythm is underdeveloped in existing literature especially in research on work. We explore these relations here through a focus on the shifting landscape of work in the UK during the COVID-19 pandemic.

Working from home in the UK due to the COVID-19 pandemic has characterised working life for many since the extraordinary events of 2020 unfolded. As a result of lockdown and social distancing measures, the rhythms of daily life were drastically and suddenly altered. Spatial rhythms changed as people worked in their domestic spaces and adapted to other people’s use of shared space, and temporal rhythms changed as different working patterns took hold. Pre-pandemic, research considered the emancipatory opportunities that a working life freed from the temporal constraints of the daily commute might offer (Lachapelle et al., 2018; Stutzer & Frey, 2008). However, during the pandemic, a majority of people in social surveys identified boredom as one of the most difficult aspects of their prolonged isolation, second only to lack of freedom (Mann, 2020). At the same time, those working from home were exhorted to not feel bored, and to check their/our privilege, as people in health and essential services continued working under adverse and often life-threatening conditions.

In this article, we analyse boredom as a feature and narrative dimension of homeworking life, based on a selection of materials (online blogs and commentaries) in the public domain which reflect on working (from home) both during the lockdowns of 2020, and the move towards a ‘new normal’ way of working in 2021. Drawing on the ‘rhythmanalytic project’ of Henri Lefebvre, specifically *Rhythmanalysis* (2004), developed with Catherine Régulier, we foreground rhythm as a lens for exploring boredom and work in these commentaries. We identify a specific form of boredom, *acedia*, a particular type of torpor or listlessness, as featuring prominently in the accounts of rhythmic rupture. The origins of the concept of acedia lie in its connection to a spiritual state, and one deemed a sin by Christian moralizers of the 4th century Ad (Wenzel, 2017). Despite the dangers of using concepts outside of their historical context, we argue that acedia resonates strongly with the paralysing boredom described or feared by those working at home under lockdown conditions.

This article does three things. First, it offers an empirical analysis of online accounts of boredom as a particular phenomenon that may have widely affected the global working population. Secondly, we make a conceptual contribution, illustrating how thinking through boredom, in particular acedia, and rhythm sheds light on working life in and beyond the COVID-19 pandemic. It also makes a methodological contribution, illustrating the way in which rhythmanalysis can be used to guide the analysis of online textual content. The article is structured as follows. We review key literature on flexible working/homeworking and boredom at work, and propose acedia in particular as relevant for making sense of boredom and work in the coronavirus pandemic. We review Lefebvre’s work on rhythms and his insights into the sociology of boredom, setting out how we make use of rhythm and rhythmanalysis conceptually and methodologically. We present our methodology then discuss our analysis around three sets of themes: stalling and stillness in the present; embodiment; and present–future relations. Overall, we open up a dialogue on the relationship between rhythm, boredom and the context of homeworking, space and time in the pandemic.

## Boredom, acedia and homeworking

In existing literature, boredom at work is analysed as arising from a combination of objective work conditions and subjective monotony (Melamed et al., 1995), from specific environments, and from a person–environment mismatch (Fisherl, 1993). Monotonous, repetitive work tasks have long been correlated with boredom (Hamper, 1991; Smith, 1981). In Roy’s classic study of time, routine and interaction at work, ‘Banana Time’ (1959), he found that play between assembly-line workers engaged in repetitive tasks absorbed their attention and punctuated the days, making them pass more quickly. As Johnsen (2016, p. 1404) describes: ‘Boredom may be viewed in terms of passivity; but it may also be explained in terms of the fidgeting, doodling, shuffling activity of both mind and body that it causes and which the announcements of Roy’s cuckoo clock come to signify.’

The shift from passivity, and an implied immobility, to movement (both embodied and cognitive) connects boredom to embodiment, a theme which we develop in this article. Indeed, as Roy (1959) found, when there is a hiatus in social interaction at work, time drags and registers at a corporeal level. Boredom stills and slows time–space and as something that suspends the body’s capacities to affect and be affected (Anderson, 2004). Bissell and Fuller (2011) conceptualise stillness as a punctuation in the flow of all things, which highlights the relationship between rhythm and pause. Paasonen points to the ‘persistent ambiguity’ of boredom such that ‘the affective flatness of boredom comes speckled with minor and major fascinations’ rather than being all-encompassing (2022, p. 152). The body therefore emerges as a site of boredom which variously accepts or rebels to force movement and emerge from the bored state (Anderson, 2004; Johnsen, 2016). As Johnsen (2016, p. 1405) puts it:

The symptoms are common enough: the feeling that time is long and will not pass; the impression that everything is always the same, as if the experience itself captures a snapshot of eternity; a bodily restlessness that becomes a sudden urge to yawn, an itch that will not be satisfied, overwhelming sleepiness; the daydreaming that forces attention away from what it was supposed to be directed at.

Johnsen also discusses the connection between boredom and the sin (in Christian terminology) of ‘acedia’. The term acedia derives from the Greek ‘lack of care’, reminding us of what we might today call sloth or apathy. For Christian monks in the 4th century ad, such as Evagrius of Pontius, acedia – or ‘the noonday demon’ due to its propensity to strike at around midday – was the most important of the deadly sins which he thought might tempt monks to abandon their religious lives:

The demon of acedia . . . is the most burdensome of all the demons. First it makes the sun seem to slow down or stop moving, so that the day appears to be fifty hours long . . . Then it makes him hate the place and his way of life and his manual work. (Evagrius, 2003, p. 93)

In their now classic study of unemployment in the village of Marienthal in the 1930s, Jahoda et al. (1972/2017) explore a disrupted sense of time and the associated steady decline into apathy (Lobo, 2018); one of the time sheets completed by one of the unemployed Marienthal inhabitants reads ‘Einstweilen wird es Mittag’ or ‘In the meantime, midday comes around’ (Jahoda et al., 1972/2017, p. vii), illustrating the connection between lassitude, time and rhythm.

For Johnsen (2016), the account of Evagrius illustrates very well how closely the sin of acedia was associated with the fiercely regulated and controlled timetable of prayer and work to which the monks adhered. It was not, however, a spiritual sin that was thought to affect monks in communities; rather it was an affliction to be borne (and conquered) by monks in isolation, whose circumstances generated a strange combination of listlessness, undirected anxiety and inability to concentrate (Zecher, 2020).

In *A Book of Silence* (2008), her account of her adoption of a hermit’s life, Sara Maitland explains how difficult it is to describe acedia, since ‘its predominant feature is a lack of affect, an overwhelming sense of blankness and an odd restless and dissatisfied boredom’ (2008, p. 108). Neither the result of an ascetic nor disciplined life, acedia seems to be connected with isolation, as it is also reported in scholars working alone, and in the prolonged isolation of convalescent patients (2008, p. 112). Whilst it is related to depression or melancholy, and denotes many of the phenomena that we might today ascribe to depression, Maitland describes it as different conditions requiring different treatments. The way to treat depression, with or without medical intervention, is generally considered to be gentleness, rest and self-care, whereas the early Christian Church was very clear that acedia could only be defeated by hard work, discipline, penance and rigour (2008, p. 113). The rigours of monastic life enable the antidote to acedia, as a specific, regulated, bodily activity is prescribed as soon as the ‘demon’ takes hold.

As a result of spatial and temporal changes in the organisation and performance of work during the pandemic, swathes of the global workforce have worked from home, encompassing a huge range of industries and jobs. This explosion of homeworking has stimulated studies concerned with worker productivity (Etheridge et al., 2020), the mass hybridisation of the workforce (Parry et al., 2021) and changing workplace geographies and inequalities (Reuschke & Felstead, 2020), particularly in relation to gender (Anderson & Kelliher, 2020; Collins et al., 2021; Feng & Savani, 2020; Hjálmsdóttir & Bjarnadóttir, 2021). What is missing from this body of research is attention to the affective dimension of homeworking, the troublesome experience of boredom and its corollary of a lack of rhythm. In this article, we draw on acedia and its genealogy because we believe that it captures discussions of the experience of ‘languishing’ when working under lockdown, and because it speaks to rhythm, or, more specifically, a lack of rhythm: spatial and temporal restrictions, an unvaried and monotonous routine, social isolation with a dread of the outside world, and an inability, or difficulty, with moving between tasks (Zecher, 2020). We turn now to a discussion of how we make use of ‘rhythmanalysis’ to make sense of boredom and work in this article, conceptually and methodologically, and what this approach yields in terms of our thematic analysis.

## Using rhythmanalysis to understand boredom and work

In Lefebvre’s essays on rhythm, some co-authored with Catherine Régulier (Lefebvre, 2004), time and space are conceived as inextricably linked. What Lefebvre characterises as linear rhythms generally emanate from human and social activities and particularly from the motions of industrial work. For Lefebvre, linearity imposes a mechanical regularity and banality on the everyday. In the unfolding of natural or cyclical rhythms, he argues, there is constant creation and re-creation, a transformation which happens naturally. By contrast, linear repetition, for example the human-created routines of work, not immersed in what Gardiner (2012) calls a cohesive and organic temporal flow, results in ‘lassitude, boredom and fatigue’ (Lefebvre & Elliot, 2005, p. 130). Boredom, however, has latent potentialities, especially as it contains within it ‘frustrated frenzies [and] unrealised possibilities’ (Lefebvre, 1995, p. 124), harbouring the seeds of transformation (Gardiner, 2012).

Lefebvre and Régulier proposed rhythmanalysis as a way of researching rhythm (Lefebvre, 2004). It has been variously claimed as a science (by Lefebvre himself), an orientation or guiding principle, a sensitising concept or research strategy (see discussion in Lyon, 2019). In recent years, rhythmanalysis has gained in popularity and been deployed to research festival spaces (Duffy et al., 2011), street performance (Simpson, 2008, 2012), mobility and place-making (Chen, 2013; Edensor & Holloway, 2008; Edensor & Larsen, 2018) and work (Borch et al., 2015; Lyon, 2016; Nash, 2020). It offers a means to analyse time, space and everyday life, revealing how multiple rhythms inform individual experiences and the patterns of collective social practices (Christiansen & Gebauer, 2019, p. 8).

Our research uses rhythmanalysis to make sense of online discussions of boredom and work during the coronavirus pandemic. Our sample consists of UK blog posts and articles which make specific reference to boredom. We view this kind of material as sitting at the intersection of public and private, thereby capturing an active process of collective meaning-making generated as the extraordinary events of 2020 were unfolding and illustrating methodologically how these dominant meanings were projected and shaped interpretation, behaviour and legitimacy (Morgan, 2020). We explore how boredom is rendered in relation to homeworking, how the spatial and temporal (rhythmic) aspects of working under lockdown condition affected people’s (embodied) experiences of or statements and assumptions about boredom. Specifically, we ask: what can attention to acedia in online blogs and articles reveal about the relationship between boredom and homeworking during the pandemic? How is the body understood as a site for grappling with boredom and work? And more generally, what is the relationship between the linear and cyclical rhythms of the present and future-imagining, and what might this mean for the settings in which we will continue to work, post-COVID?

The first data collection period took place over a six-week period in October and November 2020 when spatio-temporal restrictions remained in place in the UK, and comprises online commentaries posted between April and October 2020. A total of 44 online articles and blog posts were selected, with extracts from 27 analysed in this article (see Appendix for details). The second data collection period took place over a four-week period in August 2021, comprising online commentaries posted between April and August 2021, when the UK had emerged from lockdown, but much of the workforce was still working at home. A total of 30 articles and posts were selected, with extracts from 19 analysed in this paper (see Appendix for details). In order to select relevant material, we first identified key search terms, such as ‘boredom working from home lockdown’ and ‘working rhythms routines lockdown’ (see Appendix for full list of search terms). We selected posts according to the following criteria: the title and/or content of the post covered the exact themes indicated by the search term; and that the content involved some commentary relating to the affective dimensions of boredom.

Whilst ethnography in which the body is central as a research instrument is the most widely deployed form of rhythmanalysis, other strategies for ‘grasping’ rhythm have been used in cultural history and literary studies. We draw on these to ‘read’ rhythm in the texts we gathered (e.g. Christiansen & Gebauer, 2019). In practice, initially, we created a document with extracts from the data, which we read alongside the literature discussed earlier to identify key themes. Once general themes had been noted, we undertook line by line coding and analysis The relationship between data collection and analysis was fluid in that we began to analyse the findings during the ongoing process of collection, with frequent conversations between the authors to discuss emergent themes.

We present our analysis in three parts. The first section considers the articulation of a dislocation of time and space for work once habitual spaces of work become inaccessible and explores a sense of stalling and stillness. The second focuses on the body as a site for registering rhythm whether in the felt experience of acedia, or in normative injunctions to act through the body to combat arrhythmia. The final section considers the appeal to the future for the resolution of current disruptions to rhythm.

## Stalling, stillness and acedia: Stuck in time and space

The sluggishness of time in lockdown; the frustration and torpor of days with no end in sight. (1.10)

This extract from an online article echoes a wider experience of struggling to work under lockdown. Whilst individual circumstances differ vastly, in terms of where people work, what sort of work they are engaged in, what emerges from the online accounts is a sense of dislocation of time and space. Beyond a change of routine, an absence of the usual rhythms that punctuate the day led to a collective affect of boredom (see Paasonen, 2022). However, this does not take the form of an ‘itchy’ state but is characterised by a listlessness, evocatively described as ‘a crushing sense of the monotony of directionless time and unvarying space, making it hard to keep purpose and meaning in sight’ (1.10). In the material collected in 2020, this inertia – a stalling of movement and energy – leads to a stillness, or a punctuation in the flow of things (Bissell & Fuller, 2011) that is not calm or peaceful, but melancholic, which resonates with literary and historical accounts of acedia:

This stale boredom. The listlessness that comes from staring at the same set of walls, as the days seep into one another. Difficulty summoning any interest or energy to do anything. (1.10)

A listless kind of boredom, carelessness, apathy, disinterest and restlessness all underpinned by the slow burn of a persistent, nameless anxiety. (3.4)

Changing perceptions of time, and the apparent contraction of space when working in isolation, lead to inertia:

Confined to our homes and stripped of our daily routines, many in self-isolation have found that time has become a strange and amorphous thing that can’t be defined by a calendar. (2.1)

Difficulty summoning any interest or energy to do anything in the same old space which is closing in. (1.10)

For Newtonian Physics there was a new normal with space contracting as time slowed down. After six weeks immured, I’d have been climbing the walls had they not been closing in. (3.1)

In 2021, articles reported how a significant number of employees had experienced ‘a distorted sense of time’ whilst working from home (6.4). The ‘slipping’ of time mentioned by many (e.g. 1.1, 2.1, 1.10) is reminiscent of Loukido et al.’s (2009) observation of the centrality of time to accounts of boredom, and the perception of the present as endless. Discussing the rupture that occurred in relation to the events that usually root our lives in time and space, one poster describes how ‘life tends to a be a blur without these anchors’ (6.4). Whilst most articles reporting on surveys of homeworkers post-pandemic report a majority wanting to continue working from home at least some of the time, one survey reported that almost a third of the UK has seen an impact on their mental health when working at home: ‘It’s clear that an increased sense of isolation, a lack of movement and reduced levels of motivation have affected millions’ (9.4). Now that the ‘novelty’ of homeworking has worn off, it appears that it is the lack of new experiences and face-to-face interaction that is leading to acedia, and ‘increased mental distress’ (6.1).

We read the boredom that is expressed in these accounts of homeworking under lockdown as a reaction to isolation and the absence of rhythm that emerges from changing relations of space and time. This lassitude becomes a spiritual/mental absence: ‘It leaves us spiritually exhausted to the point of not caring’ (1.10). Describing this melancholic boredom, one poster wonders ‘what could this particular form of melancholy mean in an urgent global crisis?’ and concludes that it is ‘a sense of dislocation that somehow interferes with how we care. Towards any work that may be done within the enclosure of our own lair, we become listless and inert’ (3.3). The absence of feeling that is so vividly described in the online material is a strange combination of listlessness, undirected anxiety and inability to concentrate (Zecher, 2020), and chimes with acedia and is associated with ‘lack of care’. With linear rhythms ruptured, and cyclical rhythms conflicting with the demands of work, there is a void. Whilst repetition is necessary for rhythm, it is not sufficient. Rhythm requires difference (Lefebvre, 2004). When there is no difference, when there is no pause, but instead a cessation of rhythm, acedia takes hold. It is (almost) pure and apparently endless repetition.

## Moving against boredom: Reclaiming rhythm through the body

One blog post asks, ‘what does it feel like to feel frustration in your body?’ (2.1). For most of the online posters, it feels like tiredness, immobility, difficulty concentrating and listlessness, with a churning but vague sense of anxiety permeating all tasks. The disrupted routines and isolation of lockdown, combined with the anxiety and chaos ‘outside’, lead to what is described as ‘the blur’ of the new normal (2.2). What we can perhaps conceptualise as the ‘rhythmic shock’ of lockdown leads to a mental, physical and spiritual apathy; as one poster puts it, her life is now that of a ‘caged bird’ (1.1). This ‘locking down’ of the body leads to an arrhythmic state where rhythms are distorted and discordant making it difficult to ascertain, or feel in the body, any rhythm at all with which to punctuate the days. Indeed, Anderson (2004) argues that boredom takes place as a suspension of a body’s capacities to affect and be affected. The disruption of the body’s circadian rhythms was prominent in the 2020 material, with people discussing changing sleep patterns, with either too much sleep or disrupted sleep, and particularly vivid dreams. The connection between the rhythms of the body and our mental perceptions of space and time is evoked:

Your brain and body are adjusting to fit a new style of life that has been disrupted, . . . and this, in turn, can change your perception of time. (2.2)

The recommendation in almost all posts is to move, as these excerpts from separate commentaries illustrate:

Get back into a regular exercise routine. (3.1)

Be physically active. (1.2)

Get dressed, have a clear workspace, get out for a walk at least once a day. (2.1)

Running, walking and dancing are the preferred options:

So I am shaking myself up a bit as I often do when I feel stuck. I am looking for ways to feel energetic and alive that work right here in my own little world. Running helps. (1.3)

I turned these promenades into peregrinations; physical exercise as spiritual exercises. (3.1)

A return to cyclical rhythms is advised in many posts in order to return to a rhythmic state; speaking of nature, one blogger writes: ‘There is a mental stillness to be found in yielding to her rhythms, and just accepting their power’ (2.5).

In 2021, the focus in many of the articles is on how to incorporate hybrid working into our post-pandemic lives, meaning a blend of home and office working. Whilst one poster describes a return to the office as an experience of rhythm that is ‘familiar yet foreign’ (9.1), it is to homeworking where the advice to keep moving is directed: ‘During a lunchbreak, you can even do a quick workout or join a challenge’ (8.1), ‘get up and walk around the room’ (8.2), ‘do your yoga sun salutations . . . go for a walk at 5pm’ (7.2). The noticeable difference, however, in the 2021 data compared to the 2020 data, is the increased emphasis on access to ‘natural’, or cyclical rhythms. In the later commentaries, seemingly mindful of the mental health impact experienced by so many employees, homeworkers are exhorted to get outdoor sunlight and move outdoors to create more space for oneself, practise yoga outdoors, and notice the rhythms of nature. Many commentators predict that office space will be used for ‘curated collaborations’ (7.3), in other words, for face-to-face interactions with colleagues, leaving home working for ‘everything else’, presumably non-collaborative work.

Lefebvre (2004) makes a clear distinction between cyclical rhythms, rooted in nature and the physiological rhythms of the body which are connected to naturally occurring intervals of time, for example the sun rising or the changing of the seasons, and linear rhythms which are exterior, imposed and connected to the development of industrial capitalism. Whilst he has been criticised for romanticising agrarian ways of life, this appeal is alive in the posts we analysed. The incorporation of cyclical rhythms advised in many of the posts from 2021 – e.g. being in nature, getting enough sunlight, exercising outdoors – seems to reflect a yearning for ‘natural’ rhythms, even as we come to terms with the disruption to linear rhythms.

If rhythms are apprehended or ‘folded in and through the permeable body’ (Edensor, 2012, p. 4), the incorporation of movement is not just connected to an embodied state, but also to an emotional/spiritual orientation, underlining that acedia is a very particular type of boredom and inertia that responds to the restoration of rhythm. As we see in 3.1, turning physical exercise into spiritual exercise is recommended. Here is expressed the difference between acedia as a type of boredom arising in isolation, and depression; in response to feelings of low mood and anxiety, we might have expected posts to recommend soothing remedies of self-care or even medical intervention, but it is movement and the resultant incorporation of rhythm that is proffered to banish acedia. As one poster exhorts: ‘Stimulation will rid me of the noontide demon, and I will not miss him’ (3.3).

These findings support Anderson’s (2004) observation that boredom negates the body’s capacity to affect and be affected; when suffering from acedia, it is the lack of rhythm that immobilises and deadens us, and it is a reincorporation of rhythm that can help to banish it. Lefebvre claims that periods of disruption and crisis have origins in and effects upon rhythm (2004, p. 44), and it is in the body that this is perceived most acutely, and it is in and through the body that a rhythmic reintroduction is most keenly felt. However, the relationship between the linear and the cyclical here is different from Lefebvre’s opposition. It is the cessation of the linear rhythms of work that seem to have caused such disruption and they are missed for how they punctuate the day and shape the week. Still, what the online posts suggest and call for is a reintroduction of cyclical rhythms, or at least for some harmony between the linear and the cyclical as rhythm is reinscribed into daily working lives.

## The present and the future

One of the striking features of the online items we analyse in this article is the prevalence of lists (in 50% of the items with boredom in the title in searches 6 and 7). They are generally accompanied by numbers which themselves suggest a move in time towards the future. Reading them can feel like taking steps. First, one move, change or new practice, then another, and the promise of banishing boredom comes closer. Indeed, our tolerance of boredom appears to be low (7.2). In one case, the lists of things to do when bored includes ‘Make Lists’ (7.4). In the discussion in this section, we pay attention to three aspects of the relationship between boredom, time and rhythm. First, we highlight the present as ‘extended’ (Nowotny, 1994) or endless (Loukidou et al., 2009) in discussions of boredom. Second, we focus on the appeal of the future as a space of the resolution or eradication of boredom. Finally, we discuss the attraction of new rhythms alongside a desire for the resumption of pre-pandemic patterns of life.

The emphasis on monotony we have already discussed can be recast here as an ‘extended’ or endless present and this is our focus of the first part of this discussion. The relentlessness of boredom is an affective and embodied state that lacks rhythm – ‘experience without qualities’, as Elizabeth Goodstein (2005) puts it. We have already heard accounts of time as ‘a strange and amorphous thing’ (2.1) that has become disconnected from the linear and cyclical rhythms of the calendar, for instance ‘long days unstructured by work or school’ (6.4). At the same time, we find calls to ‘relearn the fine art of human interaction’ (9.1). This may take a more general form: ‘seek quality in the now’ (7.1). These messages call for readers to stay in the present – ‘accept it’ (6.3) – and recognise its constraints; however, they do not always appreciate the existential dimensions and challenges of boredom, the intrinsic lack of desire associated with acedia.

Second, we focus on the appeal of the future as a space of the resolution of boredom and the move to eradicate the present instead of recognising boredom as a state of ‘suspended anticipation’ (Johnsen, 2016, p. 1405). ‘Get over your boredom’, entreats one commentator (1.5); ‘overcoming boredom can be difficult’ recognises another (7.2). If that doesn’t work, there is an appeal to ‘forgetting’ and a whole host of activities to pursue (getting creative, being productive, interacting, having fun and avoiding boredom in the first place) (7.2). ‘I look forward to shaking off my acedia’, another blogger comments (3.3). Indeed, an orientation to the future, a time – and space – beyond this present is sometimes presented as a resolution; and operates as a form of anticipation which actively shapes and inhabits the present (Adams et al., 2009). ‘Plan ahead’ (7.1), ‘Plan a special event’ (7.5), ‘Plan a party’, ‘Your next trip’ or even a ‘Meal plan for the week’ (numbers 29, 58 and 61 of 97 ‘Things to do when you’re bored’, 5.1). ‘Make plans’ the advice simply states, unproblematically stepping over the challenges of the present to invest in ‘upcoming trips and events’ (7.2) or to ‘plan your next vacation down to the very last detail’ (6.2).

The emphasis here is that ‘you can map out a plan’ in quite specific ways, for instance in relation to travel where the ‘reach’ of the future (Mische, 2009) is proximate but also in a more fundamental way: ‘you can still work toward and accomplish these goals’ (7.4). Or, ‘Come up with a new plan for achieving them [your goals] and envision what it will do for you in the near future’ (7.4), or ‘dedicate future time to passion projects’ (6.2). The linearity in these perspectives is powerful. There are no lives without goals or progress towards them. If anything, this points to the difficulty of waiting and tolerating the radical uncertainty of the time of the pandemic, even if it does not recognise the unequal power relations this implies (Schwartz, 1974). For Lefebvre, the avoidance of boredom, including through self-help techniques, fails ‘because they do not go beyond the confines of our privatised and commodified life experience’ (Gardiner, 2012, p. 59).

The appeal to rhythm takes a variety of forms and this is the final focus of this part of our discussion. In some of our online materials, there is an implicit recognition that productivity is threatened by the stasis of boredom. ‘Productivity’ is appreciated as a welcome effect of exercise for instance (6.3), albeit both restricted and folded into work schedules. A taken for granted reassertion of normality is also present: ‘Sooner or later after all this, we’ll return to our usual frenzied rhythms’ says number 56 in a list of ‘100 useful things you can do to kill boredom’ (10.2). Without productivity, time is hard to measure and counts for little. The affirmation of the problem of boredom can be read as a normative assertion of the value of productivity (and consumption).

That said, there are alternative perspectives which ‘harbour flashes of subversive insight’ (Gardiner, 2012, p. 38). There is recognition of what a resumption of ‘normality’ might mean: ‘gradually picking up routines’ as well as ‘setting down’ others which have been the stuff of everyday life during lockdown (2.1). In an example discussed earlier, a blogger reports having ‘found a rhythm to our days’ (1.3) which is a return to a simpler way of life and an appreciation of emotional connection. For Lefebvre (2014), what he describes as the ‘colonisation’ of everyday life by the linear rhythms of capitalism is never total or definitive. The ‘opportunity to shift things around’ inherent in pandemic time is something advocated by some bloggers, for instance to ‘create a schedule that accommodates your rhythms instead of an employer’s’ (2.4). The injunction to ‘find another gear’ is an explicit critique and challenge to ‘outsourcing the control of our attention and time’ (6.3). And ‘just *doing nothing*’ is asserted as a ‘once in a lifetime opportunity’ (7.4). This is a position which echoes some calls ahead of the crisis of the pandemic, for instance, Josh Cohen’s (2019) book, *Not Working*. And it chimes with Lefebvre’s recognition of the transformative potential of the rupture of linear time.

## Conclusions

This article has explored how rhythmanalysis can ‘grasp’ boredom through a consideration of UK online posts during the lockdowns of 2020 and the subsequent partial return to a ‘new normal’ in 2021. It offers insight into the relationship between rhythm, work and boredom. In so doing, it adds to a field of research which uses the thinking of Henri Lefebvre as a methodological and theoretical starting point, connecting the spatial and the temporal to the ways in which work and boredom are experienced. In particular, we have focused our attention on ‘acedia’ to highlight a specific quality of boredom and the relationship with work that has emerged in this time. Whilst the linear rhythms of everyday urban working life, pre-pandemic, were thought to induce boredom and malaise (Lefebvre, 2004) as a result of overload (Mosruinjohn & Matorina, 2019–2020), it would appear that a sudden disruption to the linearity and frenetic activity has resulted in much the same. Furthermore, if boredom has been discussed for its potentialities as well as its disenchantment, acedia points to the ongoingness of undifferentiated time in the pandemic in which change and alternative patterns of living and working are difficult to conceive.

This article makes three contributions to rethinking the relations between boredom, rhythm and work. First, we make an empirical one, through our exploration of rhythm in relation to work and boredom in the context of disruption. Here we highlight the relevance of space as well as time, the significance of the body and embodied rhythms to the representation and experience of boredom, and the prominence of ‘acedia’ as a particular quality of boredom during the COVID-19 pandemic. Second, we make a conceptual contribution, illustrating how boredom, in particular acedia, can be conceptualised as a disruption and absence of rhythm in our working – and everyday – lives. Third, we make a methodological contribution by illustrating how rhythmanalysis can be used to guide the analysis of online textual content in our reading of rhythm through and across a set of materials which sit at the interface of the public and private. Overall, we argue that rhythm helps us to make sense of boredom. It takes us beyond thinking about boredom purely in relation to time (in terms of quantity or quality). Instead, by recognising the significance of space and embodiment, rhythm (both linear and cyclical rhythms) helps us reconfigure boredom, which in turn may point the way to how we reconfigure our post-pandemic working lives. If we want to avoid boredom, there is a need for imaginative solutions to reinscribe linear and cyclical rhythms and new spatio-temporal patterns and rhythmic combinations across domestic spaces and workplaces, especially if the much-discussed hybrid working involves ‘curated collaborations’ (7.3) and creative work in the office, but solitary work at home.

Whilst boredom has been conceptualised as having emancipatory possibilities, the accounts of acedia during COVID that are presented and analysed in this article do not point to the creation of a liberatory space, or one which allows us more autonomy in our working lives. It is a state that produces an experience of time which is depleted of rhythm (see Highmore, 2010). Yet in his account of modernity, Lefebvre (1995) explores how, under certain conditions, boredom can be full of desires, frustrations and possibilities. The lassitude, boredom and fatigue which arise in the human experience of modernity can, he claims, be overcome by the restoration of the shared experience of lived temporality, wherein ‘emotions, feelings and subjectivity would be reaffirmed, along with rhythm, body movements, the life of the flesh’ (Lefebvre, 1995, in Gardiner, 2012, p. 46). The ‘Great Pause’ brought about a rhythmic stasis for many homeworkers that does not as yet appear to have opened up what Lefebvre (1995) describes as the potentialities of boredom, and in fact resulted in acedia. However, if acedia is characterised by a lack of rhythm, an amorphous and timeless cloud, it does contain a form of restlessness which might also prompt a return to rhythm and the restructuring of space, time and the body. Indeed, boredom is highly contradictory and diverse claims about boredom during COVID-19 have been used to narrate the collective affective experience of the pandemic (Anderson, 2021, p. 198; Paasonen, 2022). As we return to a ‘new normal’, we recognise that the past is a selective social construction (Connerton, 1989) and the alternative rhythmic patterns that we explore in this article may be forgotten in the collective narratives of the lockdowns, commemorated or simply incorporated. Our focus on the experience of acedia, however, does open up possibilities for future research into how we might reincorporate rhythm into our working lives. Indeed, experiences of homeworking offer the potential for something shared and possibly even transformative for our future ‘hybrid’ working lives, with rhythm holding the key.

## Appendix: Data used for findings in this article (drawn from overall data set)

### Search terms

#### 2020

*1. Boredom working from home lockdown*
*2. Working rhythms routines lockdown*
*3. Acedia and lockdown*
*4. Rhythms work pandemic*
*5. Advice for boredom during the pandemic*

#### 2021

*6. Bored working from home 2021*
*7. Rhythms boredom homeworking 2021*
*8. How to combat boredom working from home*
*9. Rhythms of the new normal*
*10. Hybrid working boredom*

**Table:**

Search term	URL	Code
**1**	https://www.welcometothejungle.com/en/articles/i-hate-working-from-home	**1.1**
**1**	https://www.studentmindsblog.co.uk/2020/06/is-boredom-getting-you-down-in-lockdown.html	**1.2**
**1**	http://welshhillsagain.blogspot.com/	**1.3**
**1**	https://defradigital.blog.gov.uk/2020/04/17/coronavirus-living-alone-and-working-from-home-in-lockdown/	**1.4**
**1**	https://www.independent.co.uk/life-style/health-and-families/coronavirus-working-home-tips-outbreak-how-a9374806.html	**1.5**
**1**	https://www.yarnfieldpark.com/blog/bored-working-from-home	**1.6**
**1**	https://www.personneltoday.com/hr/give-tasks-meaning-and-lockdown-monotony-can-be-defeated/	**1.7**
**1**	https://www.bbc.co.uk/news/business-51868894	**1.8**
**1**	https://homethoughtsweekly.blogspot.com/	**1.9**
**1**	http://www.open.ac.uk/blogs/religious-studies/?p=1081	**1.10**
**2**	https://www.mentalhealth.org.uk/coronavirus/looking-after-your-mental-health-while-working-during-coronavirus	**2.1**
**2**	https://www.civilservicecollege.org.uk/news-top-tips-to-improve-your-work-routine-wellbeing-in-lockdown-271	**2.2**
**2**	https://www.bbc.com/worklife/article/20200402-how-lockdown-redefines-our-weekends	**2.3**
**2**	https://wearethecity.com/five-tips-to-maintain-a-healthy-balance-while-working-from-home-during-the-lockdown/	**2.4**
**2**	https://www.highernature.com/healthy-body/heart-health/articles/circadian-rhythm	**2.5**
**3**	https://www.rlf.org.uk/showcase/writers-in-lockdown-2/	**3.1**
**3**	https://securityboulevard.com/2020/10/covid-19-and-acedia/	**3.2**
**3**	https://www.themantle.com/philosophy/battling-noontide-demon-coronavirus-lockdown-helen-waddell	**3.3**
**3**	https://www.berwickbordersyoga.co.uk/post/acceding-to-acedia	**3.4**
**4**	https://dimensions-uk.org/news-blog-post/coronavirus-days-battle-rhythms/	**4.1**
**4**	https://theconversation.com/6-things-you-can-do-to-cope-with-boredom-at-a-time-of-social-distancing-134734	**4.2**
**5**	https://i-d.vice.com/en_uk/article/n7jk77/100-things-to-do-quarantine-home-coronavirus-self-isolation-social-distancing	**5.1**
**5**	https://wonkhe.com/blogs/rhythmandblues/	**5.2**
**5**	https://www.lifehack.org/articles/featured/10-ways-to-conquer-boredom-and-feeling-too-busy.html	**5.3**
**5**	https://www.thehomeworker.com/how-to-relieve-work-from-home-boredom-in-lockdown/	**5.4**
**5**	https://www.thread.com/gb/tips/men/lifestyle/defence-boredom/	**5.5**
**5**	https://www.grenade.com/blogs/all/7-tips-to-combat-boredom-during-lockdown	**5.6**
**6**	https://www.theguardian.com/business/2021/jul/04/revealed-rise-in-stress-among-those-working-from-home	**6.1**
**6**	https://www.forbes.com/sites/nigeldavies/2021/05/31/how-to-battle-boredom-and-help-people-love-their-jobs-again/?sh=48eca73811cc	**6.2**
**6**	https://www.peoplemattersglobal.com/blog/life-at-work/bored-to-death-while-working-from-home-29415	**6.3**
**6**	https://fortune.com/2021/03/09/covid-pandemic-how-life-has-changed-coronavirus-one-year-later-march-2020/	**6.4**
**7**	https://www.cipd.co.uk/knowledge/fundamentals/relations/flexible-working/remote-working-top-tips#gref	**7.1**
**7**	https://www.annalevycoaching.com/blog/2021/4/30/is-remote-working-getting-you-down	**7.2**
**7**	https://www.triflecreative.com/blog/what-we-missed-in-the-workspace-in-2020-and-hope-to-gain-in-2021	**7.3**
**7**	https://rhythmsofplay.com/get-organized-good-establishing-daily-rhythm/	**7.4**
**7**	https://www.flexjobs.com/blog/post/how-to-structure-your-day-when-working-from-home/	**7.5**
**8**	https://www.summit.co.uk/10-easy-ways-to-make-working-from-home-productive-and-fun/	**8.1**
**8**	https://realbusiness.co.uk/working-hometop-tips-productivity	**8.2**
**8**	https://www.william-russell.com/blog/finding-the-right-work-life-balance-when-working-from-home/	**8.3**
**9**	https://www.forbes.com/sites/servicenow/2021/08/06/the-art-of-being-human-in-the-new-world-of-work/?sh=2732d8371a5b	**9.1**
**9**	https://www.managers.org.uk/knowledge-and-insights/article/how-to-work-with-your-people-to-shape-the-new-normal/	**9.2**
**9**	https://www.cipd.co.uk/knowledge/fundamentals/relations/flexible-working/planning-hybrid-working#gref	**9.3**
**9**	https://wearewildgoose.com/uk/news/the-2021-working-from-home-survey/	**9.4**
**9**	https://www.refinery29.com/en-gb/burnout-working-from-home-how-to-manage	**9.5**
**10**	https://fridaypulse.com/boredom-is-a-job-killer-we-need-an-interest-stimulus/	**10.1**
**10**	100 useful things to do when bored in quarantine - i-D (http://vice.com)	**10.2**

## References

[bibr1-00380261221147749] Adams V. Murphy M. Clarke A. E. (2009). Anticipation: Technoscience, life, affect, temporality. Subjectivity, 28, 246–265.

[bibr2-00380261221147749] Anderson B. (2004). Time-stilled space-slowed: How boredom matters. Geoforum, 35(6), 739–754.

[bibr3-00380261221147749] Anderson B. (2021). Affect and critique: A politics of boredom. Society and Space, 39(2), 197–217.

[bibr4-00380261221147749] Anderson D. Kelliher C. (2020). Enforced remote working and the work–life interface during lockdown. Gender in Management: An International Journal, 35(7/8), 677–683.

[bibr5-00380261221147749] Bissell D. Fuller G. (Eds.). (2011). Stillness in a mobile world. Routledge.

[bibr6-00380261221147749] Borch C. Hansen K. B. Lange A. C. (2015). Markets, bodies, and rhythms: A rhythmanalysis of financial markets from open-outcry trading to high-frequency trading. Environment and Planning D: Society and Space, 33(6), 1080–1097.

[bibr7-00380261221147749] Boylan J. Seli P. Scholer A. A. Danckert J. (2021). Boredom in the COVID-19 pandemic: Trait-boredom proneness, the desire to act, and rule-breaking. Personality and Individual Differences, 17(1), 1–6. 10.1016/j.paid.2020.110387 PMC9045809 35502306

[bibr8-00380261221147749] Carroll B. J. Parker P. Inkson K. (2010). Evasion of boredom: An unexpected spur to leadership? Human Relations, 63(7), 1031–1049.

[bibr9-00380261221147749] Chen Y. (2013). ‘Walking with’: A rhythmanalysis of London’s East End. Culture Unbound, 5(4), 531–549.

[bibr10-00380261221147749] Christiansen S. L. Gebauer M. ( 2019). Lefebvre and rhythms today.In Christiansen S. L. Gebauer M. (Eds.), Rhythms now: Henri Lefebvre’s rhythmanalysis revisited [Open Access ed.]. Aalborg Universitetsforlag. Interdisciplinære kulturstudier: 2709743_Rhythm_165x235_ONLINE.indd (aau.dk)

[bibr11-00380261221147749] Cohen J. (2019). Not working: Why we have to stop. Granta Books.

[bibr12-00380261221147749] Collins C. Ruppanner L. Landivar L. C. Scarborough W. J. (2021). The gendered consequences of a weak infrastructure of care: School reopening plans and parents’ employment during the COVID-19 pandemic. Gender & Society, 35(2), 180–193.

[bibr13-00380261221147749] Connerton P. (1989). How societies remember. Cambridge University Press.

[bibr14-00380261221147749] Costas J. Kärreman D. (2016). The bored self in knowledge work. Human Relations, 69(1), 61–83.

[bibr15-00380261221147749] Dag Holmboe R. Morris S. (Eds.). (2021). On boredom: Essays in art and writing. UCL Press.

[bibr16-00380261221147749] Duffy M. Waitt G. Gorman-Murray A. Gibson C. (2011). Bodily rhythms: Corporeal capacities to engage with festival spaces. Emotion, Space and Society, 4(1), 17–24.

[bibr17-00380261221147749] Edensor T. (Ed.). (2012). Geographies of rhythm: Nature, place, mobilities and bodies. Ashgate.

[bibr18-00380261221147749] Edensor T. Holloway J. (2008). Rhythmanalysing the coach tour: The Ring of Kerry, Ireland. Transactions of the Institute of British Geographers, 33(4), 483–501.

[bibr19-00380261221147749] Edensor T. Larsen J. (2018). Rhythmanalysing marathon running: ‘A drama of rhythms’. Environment and Planning A, 50(3), 730–746.

[bibr20-00380261221147749] Etheridge B. Tang L. Wang Y. (2020). Worker productivity during lockdown and working from home: Evidence from self-reports. Covid Economics, 1(52), 118–151.

[bibr21-00380261221147749] Evagrius P. (2003). Eulogios ( Sinkewicz R. , Trans.). Oxford University Press.

[bibr22-00380261221147749] Feng Z. Savani K. (2020). Covid-19 created a gender gap in perceived work productivity and job satisfaction: Implications for dual-career parents working from home. Gender in Management: An International Journal, 35(7/8), 719–736.

[bibr23-00380261221147749] Fisherl C. D. (1993). Boredom at work: A neglected concept. Human Relations, 46(3), 395–417.

[bibr24-00380261221147749] Gamsby P. (2018). Boredom: Emptiness in the modern world.In Jacobsen M. H. (Ed.), Emotions, everyday life and sociology (pp. 209–224). Routledge.

[bibr25-00380261221147749] Gardiner M. E. (2012). Henri Lefebvre and the ‘sociology of boredom’. Theory, Culture & Society, 29(2), 37–62.

[bibr26-00380261221147749] Gardiner M. E. Haladyn J. J. (Eds.). (2016). Boredom studies reader: Frameworks and perspectives. Routledge.

[bibr27-00380261221147749] Goodstein E. (2005). Experience without qualities: Boredom and modernity. Stanford University Press.

[bibr28-00380261221147749] Hamper B. (1991). Rivethead: Tales from the assembly line. Warner.

[bibr29-00380261221147749] Healy S. D. (1984). Boredom, self, and culture. Fairleigh Dickinson University Press.

[bibr30-00380261221147749] Highmore B. (2010). Ordinary lives: Studies in the everyday. Routledge.

[bibr31-00380261221147749] Hjálmsdóttir A. Bjarnadóttir V. S. (2021). ‘I have turned into a foreman here at home’: Families and work–life balance in times of COVID-19 in a gender equality paradise. Gender, Work & Organization, 28(1), 268–283. 33041540 10.1111/gwao.12552 PMC7537149

[bibr32-00380261221147749] Jahoda M. Lazarsfeld P. F. Zeisel H. Fleck C. (2017). Marienthal: The sociography of an unemployed community. Routledge (Original work published 1972).

[bibr33-00380261221147749] Johnsen R. (2016). Boredom and organization studies. Organization Studies, 37(10), 1403–1415.

[bibr34-00380261221147749] Lachapelle U. Tanguay G. A. Neumark-Gaudet L. (2018). Telecommuting and sustainable travel: Reduction of overall travel time, increases in non-motorised travel and congestion relief? Urban Studies, 55(10), 2226–2244.

[bibr35-00380261221147749] Lefebvre H. (1995). Introduction to modernity ( Moore J. , Trans.). Verso.

[bibr36-00380261221147749] Lefebvre H. (2004). Rhythmanalysis: Space, time and everyday life. A&C Black.

[bibr37-00380261221147749] Lefebvre H. (2014). Critique of everyday life: The one-volume edition. Verso (Original works published 1947, 1961, 1981).

[bibr38-00380261221147749] Lefebvre H. Elliot G. (2005). Critique of everyday life. Vol. 3, From modernity to modernism (Towards a metaphilosophy of daily life). Verso.

[bibr39-00380261221147749] Lobo F. (2018). Unemployment and leisure: The Marienthal legacy. World Leisure Journal, 60(2), 75–93.

[bibr40-00380261221147749] Loukidou L. Loan-Clarke J. Daniels K. (2009). Boredom in the workplace: More than monotonous tasks. International Journal of Management Reviews, 11(4), 381–405.

[bibr41-00380261221147749] Lyon D. (2016). Doing audio-visual montage to explore time and space: The everyday rhythms of Billingsgate Fish Market. Sociological Research Online, 21(3), 57–68.

[bibr42-00380261221147749] Lyon D. (2019). What is rhythmanalysis? Bloomsbury Publishing.

[bibr43-00380261221147749] Maitland S. (2008). A book of silence: A journey in search of the pleasures and powers of silence. Granta Books.

[bibr44-00380261221147749] Mann M. (2020, May 20). Don’t fight the boredom. The Atlantic. http://www.theatlantic.com/family/archive/2020/05/why-you-should-embrace-boredom-lockdown/611392/

[bibr45-00380261221147749] Melamed S. Ben-Avi I. Luz J. Green M. S. (1995). Objective and subjective work monotony: Effects on job satisfaction, psychological distress, and absenteeism in blue-collar workers. Journal of Applied Psychology, 80(1), 29–42. 7706193 10.1037/0021-9010.80.1.29

[bibr46-00380261221147749] Mische A. (2009). Projects and possibilities: Researching futures in action. Sociological Forum, 24(3), 694–704.

[bibr47-00380261221147749] Morgan M. (2020). Why meaning-making matters: The case of the UK Government’s COVID-19 response. American Journal of Cultural Sociology, 8(3), 270–323. 33078075 10.1057/s41290-020-00121-y PMC7557151

[bibr48-00380261221147749] Mosruinjohn S. Matorina N. (2019–2020). For boredom: A conceptual network of cultural objects. Capacious: Journal for Emerging Affect Inquiry, 2(1–2). http://capaciousjournal.com/past-issues/vol-2-nos-1-2-2019-2020/

[bibr49-00380261221147749] Nash L. (2020). Performing place: A rhythmanalysis of the city of London. Organization Studies, 41(3), 301–321.

[bibr50-00380261221147749] Nowotny H. (1994). Time: The modern and postmodern experience. Polity Press.

[bibr51-00380261221147749] Paasonen S. (2022). Experimentations in pandemic boredom. In Timm Knudsen B. Krogh M. Stage C. (Eds.), Methodologies of affective experimentation (pp. 139–157). Palgrave Macmillan.

[bibr52-00380261221147749] Parry J. Young Z. Bevan S. Veliziotis M. Baruch Y. Beigi M. Bajorek Z. Salter E. Tochia C. (2021). Working from home under COVID-19 lockdown: Transitions and tensions. University of Southampton. eprints.soton.ac.uk/446405/

[bibr53-00380261221147749] Reuschke D. Felstead A. (2020). Changing workplace geographies in the COVID-19 crisis. Dialogues in Human Geography, 10(2), 208–212.

[bibr54-00380261221147749] Roy D. (1959). ‘Banana time’: Job satisfaction and informal interaction. Human Organization, 18(4), 158–168.

[bibr55-00380261221147749] Schwartz B. (1974). Waiting, exchange, and power: The distribution of time in social systems. American Journal of Sociology, 79(4), 841–870.

[bibr56-00380261221147749] Simpson P. (2008). Chronic everyday life: Rhythmanalysing street performance. Social & Cultural Geography, 9(7), 807–829.

[bibr57-00380261221147749] Simpson P. (2012). Apprehending everyday rhythms: Rhythmanalysis, time-lapse photography, and the space-times of street performance. Cultural Geographies, 19(4), 423–445.

[bibr58-00380261221147749] Smith R. P. (1981). Boredom: A review. Human Factors, 23(3), 329–340. 7016721 10.1177/001872088102300308

[bibr59-00380261221147749] Stutzer A. Frey B. S. (2008). Stress that doesn’t pay: The commuting paradox. Scandinavian Journal of Economics, 110(2), 339–366.

[bibr60-00380261221147749] Wenzel S. (2017). The sin of sloth: Acedia in medieval thought and literature. University of North Carolina Press.

[bibr61-00380261221147749] Zecher J. (2020, August 27). Acedia: The lost name for the emotion we’re all feeling right now. The Conversation. https://theconversation.com/acedia-the-lost-name-for-the-emotion-were-all-feeling-right-now-144058

